# Technical tips and tricks for complex biplanar high tibial osteotomies

**DOI:** 10.1002/ksa.70083

**Published:** 2025-09-29

**Authors:** Tyler M. Hauer, Romed P. Vieider, David Wasserstein, Matthieu Ollivier, Volker Musahl

**Affiliations:** ^1^ Department of Orthopaedic Surgery, UPMC Freddie Fu Sports Medicine Center University of Pittsburgh Pittsburgh Pennsylvania USA; ^2^ Division of Orthopaedic Surgery University Health Network University of Toronto Toronto Ontario Canada; ^3^ Department of Sports Orthopaedics, TUM University Hospital Technical University of Munich Munich Germany; ^4^ Schatzker Joint Preservation Initiative – Division of Orthopedic Surgery, Sunnybrook Health Sciences Centre University of Toronto Toronto Ontario Canada; ^5^ Aix Marseille Univ, CNRS, ISM, Inst Movement Sci Marseille France

**Keywords:** high tibial osteotomy, knee, medial opening wedge, osteoarthritis, osteotomy, posterior tibial slope

## Abstract

**Purpose:**

While traditional high tibial osteotomy (HTO) techniques primarily address malalignment in the coronal plane, the significance of sagittal plane alignment, particularly the posterior tibial slope (PTS), is not to be overlooked in the setting of cruciate ligament insufficiency. Combined deformities involving both the coronal plane and the sagittal plane are less common and present unique surgical challenges. This narrative review summarizes the literature and introduces tips and tricks for managing complex biplanar deformities through a case‐based discussion of different techniques.

**Methods:**

This narrative review includes preoperative planning, surgical techniques, clinical outcomes and illustrative clinical cases detailing surgical rationale and technical nuances in the correction of biplanar proximal tibial deformities. Emphasis is placed on the importance of accurate assessment and correction of biplanar deformities to optimize patient outcomes. Four representative technique presentations are included: (1) Hybrid HTO with a posterior opening wedge (POW) and anterior closing wedge (ACW), (2) asymmetrical medial closing wedge (MCW) HTO, (3) medial opening wedge (MOW) HTO with an anterolateral hinge and (4) a double HTO with both an infratuberosity ACW and high MOW.

**Conclusion:**

Biplanar HTO is a knee‐preserving surgical option for a small cohort of patients with complex knee deformities involving both the coronal and sagittal planes. Precise preoperative planning and meticulous surgical execution are essential to address these biplanar malalignments effectively. This narrative review serves as a guide for orthopaedic surgeons, highlighting key considerations when planning biplanar HTO and serves as a practical guide for complex cases.

**Level of Evidence:**

Level V.

**Abbreviations**
3D: three‐dimensional
ACL: anterior cruciate ligament
ACW: anterior closing wedge
CT: computed tomography
HTO: high tibial osteotomy
JLCA: joint line convergence angle
LCW: lateral closing wedge
LDFA: lateral distal femoral angle
LET: lateral extraarticular tenodesis
MCW: medial closing wedge
MOW: medial opening wedge
MPTA: medial proximal tibial angle
OA: osteoarthritis
PCL: posterior cruciate ligament
POW: posterior opening wedge
PSI: patient‐specific instrumentation
PTS: posterior tibial slope
SATT: static anterior tibial translation

## INTRODUCTION

High tibial osteotomy (HTO) is a powerful and well‐established procedure in knee preservation surgery [9, 36, 62]. The most common indication for HTO is varus correction in the coronal plane [57, 70], delaying or avoiding the need for total knee arthroplasty (TKA) [16, 68]. HTO can be utilized to correct coronal plane malalignment via medial opening wedge (MOW) or lateral closing wedge (LCW) techniques [26].

In addition to the coronal plane, sagittal plane malalignment can influence knee kinematics [48], joint stability [42], as well as ligamentous and meniscal loading [69]. From a biomechanical perspective, an increased posterior tibial slope (PTS) leads to greater anterior translation of the tibia in both anterior cruciate ligament (ACL) deficient and ACL‐reconstructed knees, increasing the forces and stress on the ACL graft [2, 4, 31, 33]. A PTS of >12° has been discussed as a risk factor for graft failure following ACL reconstruction [3, 54, 65, 66]. A PTS‐reducing osteotomy through an anterior closing wedge (ACW) HTO is therefore considered in revision ACL reconstruction with a PTS > 12° [12].

The PTS is also of biomechanical and clinical interest in patients with concurrent posterior cruciate ligament (PCL) insufficiency or PCL graft failure following PCL reconstruction [23, 30, 31, 67, 69]. A PTS increasing osteotomy through an anterior opening wedge HTO is considered in patients with PCL insufficiency and a PTS < 4°. In clinical practice, the PTS is evaluated preoperatively using lateral knee radiographs [3, 22, 29, 38, 64]. As a general guide, PTS can be measured off the medial plateau using perfect lateral knee radiographs with a minimum included proximal tibial shaft length of 15 cm.

Not uncommonly, biplanar deformities are present and are challenging to treat due to simultaneous three‐dimensional (3D) correction in two planes. Various techniques have been published utilizing different strategies to correct biplanar malalignment, often in two stages [13, 28, 30, 43, 49]. This review will provide a structured case‐based description of current single‐stage techniques used for the combined correction of coronal and sagittal malalignment in HTO. In four illustrative clinical cases, the surgical rationale and technical nuances associated with each approach are presented. Each of the cases was performed by one of three international experts in the field. The objective of this review is to provide a practical reference for orthopaedic surgeons faced with biplanar tibial deformities, highlighting key considerations in both the planning and execution of advanced HTO techniques.

## PREOPERATIVE PLANNING: KEY ANGULAR PARAMETERS

Preoperative planning for HTO demands a precise evaluation of coronal and sagittal alignment, deformity origin, and joint line orientation [32, 51]. High‐quality, weight‐bearing bilateral long‐leg radiographs remain the gold standard for assessing lower limb alignment [10]. Key angles include the medial proximal tibial angle (MPTA), normally between 85° and 90°, and the mechanical lateral distal femoral angle (LDFA), typically between 84° and 90° [32]. Joint line obliquity (JLO), defined as the angle between the tibial plateau and the horizontal line, should be minimized to <4–6° after correction [55]. The joint line convergence angle (JLCA), ideally between 0° and 3°, reflects coronal deformity that originates intraarticularly and may increase as degenerative chondral wear progresses [37, 44]. The named angles determine the extent of deformity and guide osteotomy planning. Advanced 3D imaging such as computed tomography (CT) may further characterize complex deformities or intra‐articular pathology and can further be used for patient‐specific instrumentation (PSI) planning [19, 40]. The authors prefer two‐dimensional planning according to the Miniaci method [41]. Preoperative planning targets the weight‐bearing line at 50%–55% (rarely to 62.5%) of the tibial plateau width, ensuring joint preservation, optimal biomechanics and decreased risk of introducing the opposite deformity, through precise correction [32].

For assessment of alignment in the sagittal plane, the authors recommend a true weight‐bearing lateral tibial radiograph involving at least 15 cm of the proximal tibia to determine the tibial mechanical axis and reliably measure the tibial slope with respect to the medial tibial plateau [18, 25, 27, 45, 64]. There is a twofold effect of PTS reducing osteotomy in ACL‐deficient knees: first, the PTS itself is reduced; and second, the static anterior tibial translation (SATT) is also decreased. The SATT refers to the relative anterior displacement of the proximal tibia with respect to the femur in the sagittal plane, and values of ≥6 mm are predictive for ACL and ACLR injury [46]. Correcting the PTS to a target angle of 4°–6° achieves the most effective balance [8]. Undercorrection may result in an insufficient effect on SATT and may not reduce forces on the ACL graft [8], whereas overcorrection (PTS < 4°) may increase the risk of PCL strain, although the clinical significance of this finding remains uncertain [8, 23]. Therefore, the authors suggest correcting the PTS to 4°–6°.

ACW‐HTO to reduce the PTS can be performed at different levels: supratuberosity, transtuberosity, or infratuberosity. Concerns about clinically relevant changes in patellar height with supratuberosity ACW‐HTO have not been confirmed in the literature, and all levels of correction have shown favourable outcomes, whereas the supra‐ and infratuberosity approaches represent the most frequently used methods [33, 50, 61].

Preoperative planning of a biplanar osteotomy affords distinctive planning and understanding of the 3D position of the hinge [47]. During MOW‐HTO, varying hinge positions and hinge axis rotation may be a reason for unintentional PTS changes during HTO but may also serve to correct the coronal and sagittal planes simultaneously [17, 58]. In MOW‐HTO, distalization and flexion of the hinge or a posterolateral hinge location can lead to stepwise increases in PTS. Similarly, proximalization and extension of the hinge or an anterolateral hinge location can lead to decreases in the PTS [17]. The sagittal inclination angle of the HTO may also influence PTS correction, as an anteriorly inclined osteotomy cut (relative to the tibial joint line) may result in an elevated PTS post‐operatively [35]. It is also important to note that numerous biomechanical and radiographic studies have demonstrated that MOW‐HTO tends to increase the PTS, whereas LCW‐HTO tends to decrease the PTS [17, 35, 49, 62]. These changes are especially pronounced in cases involving large angular corrections or when hinge position and osteotomy orientation are not meticulously controlled [17, 35].

Ultimately, technical nuances underscore the need for precise preoperative planning and intraoperative execution, particularly in patients requiring biplanar correction. Attention to hinge axis orientation, wedge asymmetry, and osteotomy level is critical to minimize unintended slope changes and optimize patient outcomes in both coronal and sagittal alignment correction [17, 35, 49, 58].

To summarize, biplanar correction might be considered in the revision setting when patients present with a combination of symptomatic varus or valgus malignment over >5° combined with an increased PTS > 12° and concomitant ACL insufficiency. Conventional planning on radiographs or 3D imaging for PSI may be considered.

## CASE 1: BIPLANAR CORRECTION—COMBINED POSTERIOR OPENING WEDGE (POW) AND ACW‐HTO TO CORRECT VARUS AND PTS USING A CENTRAL HINGE

### Clinical history

A 21‐year‐old male presented with chronic right knee pain and instability. There was a history of trauma to the right knee that occurred 4 years prior, which was followed by an ACL reconstruction with hamstring autograft and lateral extraarticular tenodesis (LET) in the same year. Two years following surgery, he began experiencing ongoing medial knee pain as well as instability during pivoting activities that did not resolve with non‐operative measures. The patient reported being able to run and participate in sports; however, these activities invariably led to knee swelling and pain. On physical examination, the patient demonstrated a negative Lachman test, a positive pivot shift test and medial joint line tenderness.

### Radiographs and planning of correction

**Figure 1:**
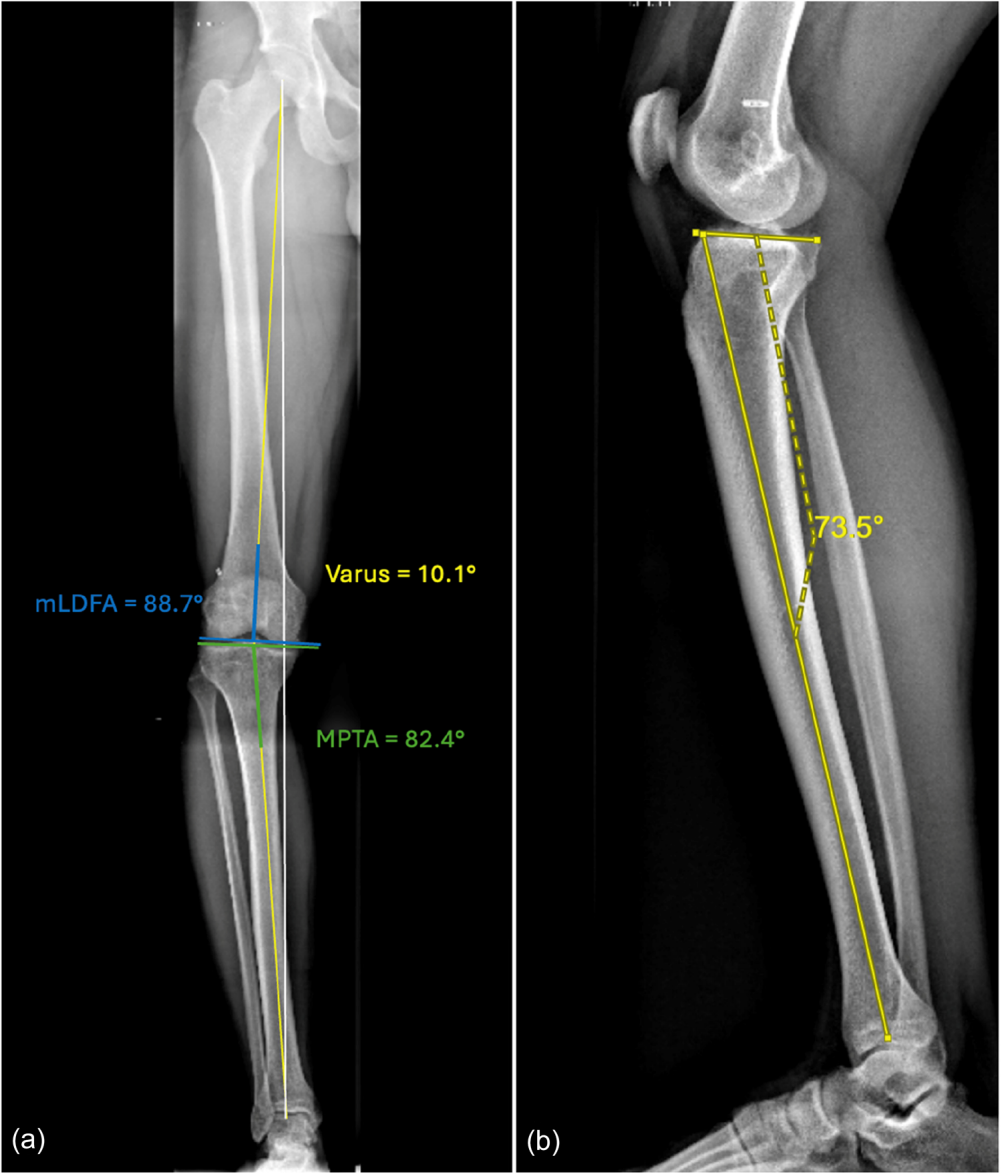
Preoperative long cassette radiographs (a) show a 10° overall varus from the proximal tibia with a medial proximal tibial angle (MPTA) of 82° and a lateral distal femoral angle (mLDFA) of 89°. In the sagittal plane, the lateral radiograph (b) demonstrates an elevated posterior tibial slope of 17°.

**Figure 2:**
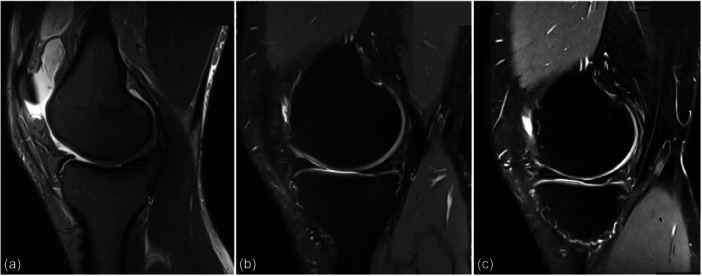
T2 sequence of a sagittal MRI of the right knee performed at different time points with (a) at symptom onset, (b) 12 months after symptom onset and (c) 2 years after symptom onset. The medial meniscus shows increasing signal intensity in the posterior horn throughout the different time points. MRI, magnetic resonance imaging.

Long cassette radiographs showed a 10° varus from the proximal tibia with an MPTA of 82° and an LDFA of 89°; in the sagittal plane, the lateral radiograph demonstrated an elevated PTS of 17° (Figure 1). Magnetic resonance imaging (MRI) demonstrated increased signal in the posterior horn of the medial meniscus and an intact ACL graft (Figure 2).

### Assessment and plan

This patient presented with a primarily tibial‐based varus malalignment warranting consideration of realignment osteotomy to offload the symptomatic medial compartment. With a PTS of 17° in the setting of an intact ACL reconstruction with LET and remaining rotational instability, the PTS should be corrected simultaneously. This case demonstrates a hybrid infratuberosity ACW combined with a POW‐HTO to correct both varus and PTS around a central hinge point.

### Surgical planning

**Figure 3:**
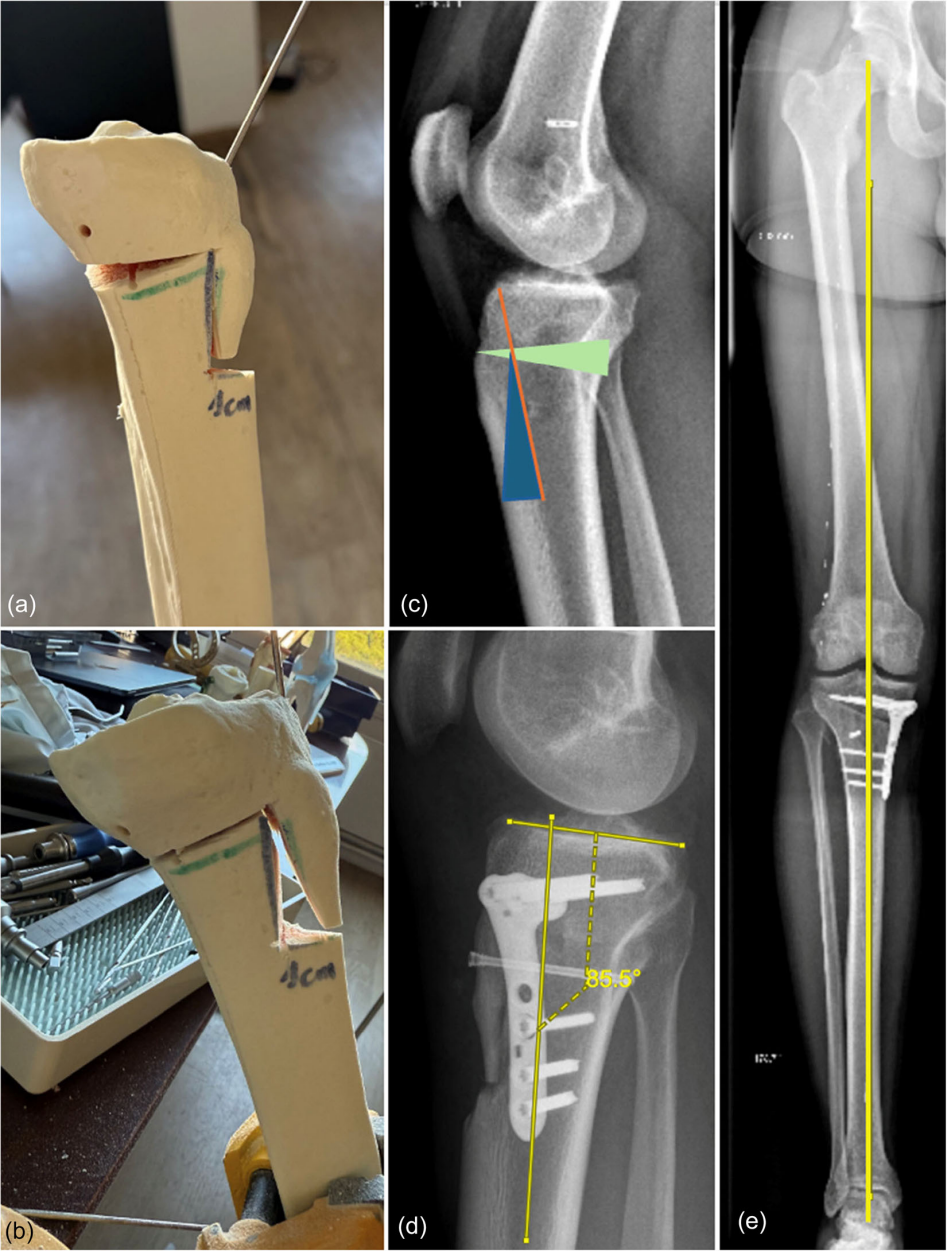
Preoperative planning for a biplanar high tibial osteotomy (HTO) to correct varus malalignment and posterior tibial slope (PTS) on a bone model (a, b). Biplanar correction is performed through a combined infratuberosity anterior closing wedge and a posterior opening wedge HTO through a central hinge point (c). By opening the wedge in an asymmetric fashion (elevated posterior and tilted anteriorly), both planes can be corrected. Post‐operative long cassette radiographs show the corrected coronal alignment (e) and the corrected PTS to 4.5° (d).

All aspects of the correction were meticulously planned using both calibrated long‐leg weight‐bearing radiographs and a 3D model due to the severe biplanar deformity. A hybrid HTO was planned combining POW to correct varus and ACW to correct PTS using a central hinge point behind the tibial tubercle. The rotational correction around the central hinge axis makes it impractical to address isolated coronal and sagittal plane deformities and underscores the need to conceptualize the osteotomy as a unified spatial correction rather than a series of independent planar adjustments. One key consideration involves the infratubercle cut since it plays a critical role in shaping the final tibial slope. The geometry of the created wedge determines the amount of PTS correction, and the anticipated bone resection may be substantial (Figure 3). Caution must be exercised to avoid planning excessive bone loss, and the authors recommend not to compromise more than 50% of the tibial cross‐section.

### Surgical steps and pearls

An anteromedial approach was used, beginning at the level of the joint line and extending distally approximately 10 cm. Care was taken to identify any residual hardware or fixation from the previous ACL reconstruction, particularly around the tibial footprint, as this could influence the osteotomy path or instrumentation.

Surgical correction began with the placement of a guide K‐wire at the predetermined hinge point, as planned on calibrated preoperative radiographs. This hinge position served as the reference for all subsequent cuts. The distal endpoint of the descending, infratubercle cut was then defined and marked, taking care to assure tubercle integrity while allowing for proper wedge geometry.

Two primary osteotomy cuts were then executed: first, the posterior cortex was cut from medial to lateral towards the hinge; second, the descending cut was made from its final point just beneath the tibial tubercle up to the hinge. With both cuts established, attention turned to the distal step, located posterior to the tibial tubercle. This was followed by an ascending cut aimed at resecting the planned retrotubercle wedge, again terminating precisely at the hinge point.

The completeness of the medial‐to‐lateral proximal cut was confirmed before any attempt at opening the osteotomy. The osteotomy was then carefully opened posteriorly, gradually creating the planned correction gap. Simultaneously, the anterior wedge was compressed using a surgical clamp, ensuring full closure without placing undue stress on the tibial tubercle, which was preserved as a structural anchor.

Once the target angular correction and anterior wedge closure were obtained, an anterior‐to‐posterior lag screw was inserted to stabilize and compress the anterior cortex. A medial locking plate was then positioned to secure the osteotomy, spanning the posteromedial gap. This gap can be filled with autograft, allograft, or bone substitute to support healing and maintain mechanical integrity.

### Post‐operative course and outcomes

Post‐operatively, the patient was placed in a PCL brace for 9 weeks to protect the correction and minimize stress on the healing osteotomy site. Non‐weight bearing (NWB) was maintained for the first 6 weeks, followed by partial weight bearing (PWB) for an additional 3 weeks, with progressive loading guided by clinical and radiographic evidence of healing. Return to daily activities and sedentary work was permitted at approximately 3 months post‐operatively, contingent on satisfactory radiographic consolidation and clinical recovery. A graduated return to sport‐specific activities—including swimming, cycling and running—was initiated at 4 months, emphasizing low‐impact, closed‐chain exercises during early reconditioning.

Two years after surgery, the patient had successfully returned to impact sports, including judo and soccer. Symptomatic hardware removal was performed. The patient's subjective knee value showed marked improvement, rising from 50% preoperatively to 90% post‐operatively.

## CASE 2: BIPLANAR CORRECTION—MEDIAL CLOSING WEDGE (MCW) HTO TO CORRECT VALGUS AND PTS

### Clinical history

A 17‐year‐old male presented with increasing instability in his right knee. Past surgical history included an ACL reconstruction with hamstring autograft 5 years prior on the same knee. The patient reported that he was never truly satisfied after the index ACL reconstruction, with persistent instability and continued pain along the lateral side of his knee. Clinical examination revealed a positive Lachman test, Grade 3 pivot shift test, lateral joint line tenderness and a dynamic valgus gait deformity.

### Radiographs and planning of correction

**Figure 4:**
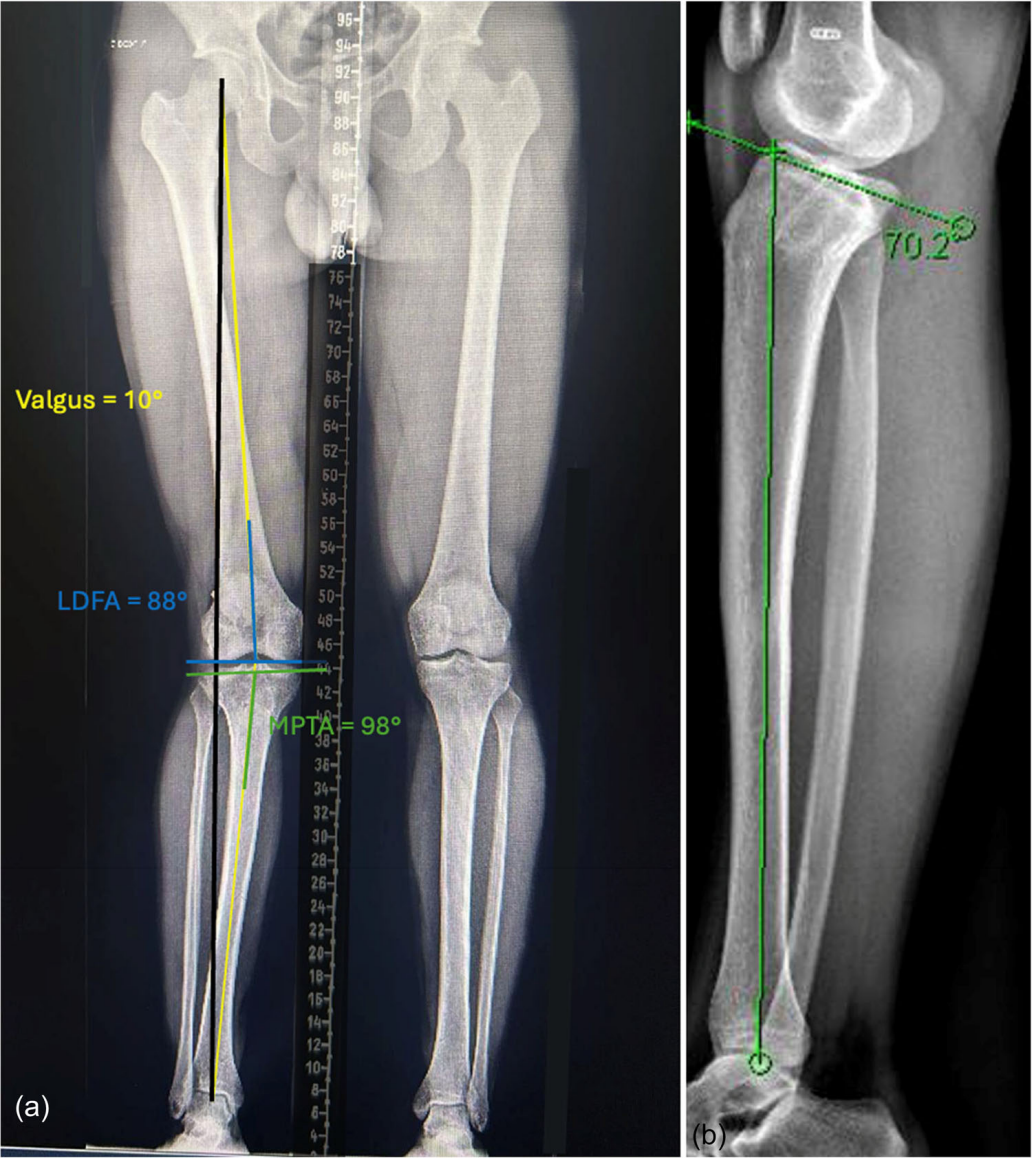
Long cassette radiographs (a) demonstrating an overall 10° valgus of the right knee. The lateral radiograph (b) demonstrates a posterior tibial slope of 20°. LDFA, lateral distal femoral angle; MPTA, medial proximal tibial angle.

**Figure 5:**
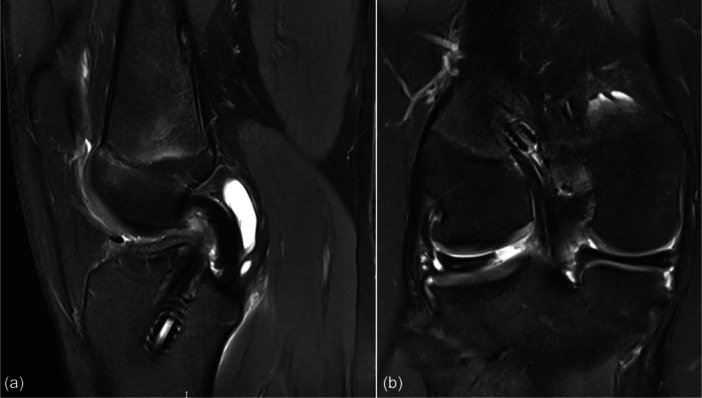
Sagittal magnetic resonance imaging of a right knee 5 years after anterior cruciate ligament (ACL) reconstruction using hamstring autograft, showing the tibial tunnel posterior to the native ACL insertion (a). The single coronal cut reveals a tear in the posterior root of the lateral meniscus (b).

The PTS measured 20° on the lateral radiograph (Figure 4b). Long cassette weight‐bearing AP radiographs revealed a mechanical valgus alignment of 10°, with a MPTA of 98°, JLCA of 2° and a LDFA of 88° (Figure 4a). MRI (Figure 5) demonstrated an intact ACL reconstruction with a posteriorly positioned tibial tunnel and a vertical graft, a medial meniscus ramp lesion and a tear of the lateral meniscus posterior root.

### Assessment and plan

**Figure 6:**
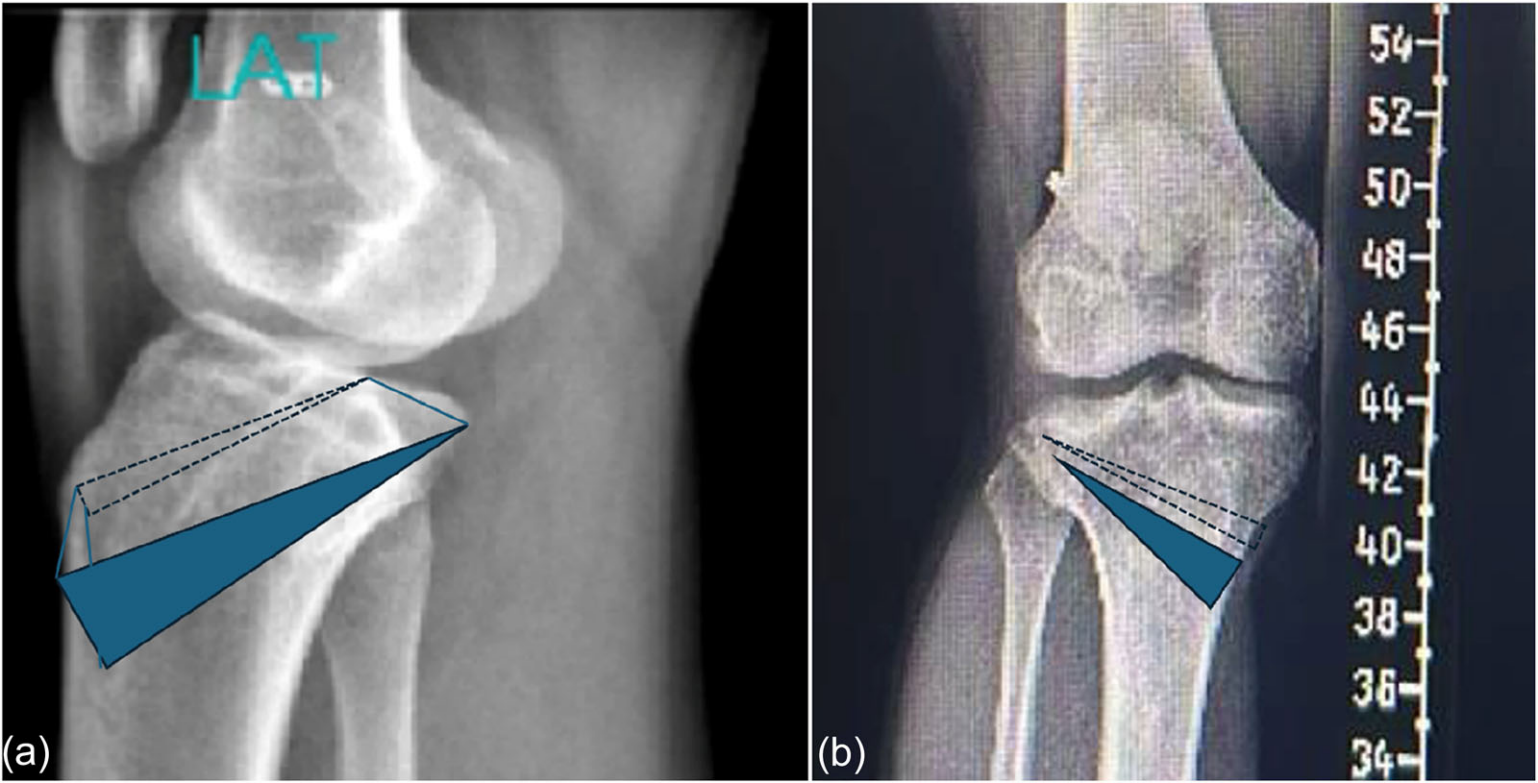
Secondary to the biplanar deformity involving both elevated valgus and posterior tibial slope (PTS), a combination of sagittal and coronal correction was planned (a, b). The planned biplanar osteotomy was performed leaving a posterolateral hinge intact to perform an asymmetric medial closing wedge and anterior closing wedge high tibial osteotomy to correct both valgus and PTS.

In the setting of valgus malalignment and an elevated PTS, realignment osteotomy to offload the lateral compartment and reduce the PTS was planned through a biplanar MCW‐HTO (Figure 6). A revision ACL reconstruction using quadriceps tendon autograft, LET, lateral meniscus root repair and a repair of the medial meniscus ramp lesion was also planned.

### Surgical planning

Preoperative planning was performed to determine the optimal hinge position, which in this case needed to be located more posteriorly and laterally to enable simultaneous closure of both the anterior and medial cortices. Two geometric triangles were constructed on the planning images to define the required closing wedges. The first triangle represented the PTS correction, with a resection planned in the retrotubercle region (Figure 6a). The second triangle outlined the MCW necessary to correct the valgus deformity (Figure 6b). Accurate execution of both wedge resections was essential to achieve correction in both the sagittal and coronal planes, while preserving the structural integrity of the tibial tubercle and maintaining hinge stability.

### Surgical steps and pearls

An anteromedial approach was performed, beginning at the level of the joint line and extending approximately 10 cm distally. The residual ACL fixation at the proximal tibia was identified to prevent interference with instrumentation or resection planes. Two K‐wires were first inserted anteromedially, aligned according to the planned anterior wedge resection for slope correction. The K‐wires were positioned to run parallel in the sagittal plane and converge laterally just above the fibular head on the AP view. On the lateral view, their intersection point was positioned close to the joint surface to ensure an appropriate posterior slope correction. The orientation of these wires intentionally targeted a more lateral hinge point than standard osteotomies, approaching the level of the fibular head rather than the PCL insertion.

Following fluoroscopic confirmation of correct positioning, two horizontal cuts were made distal to proximal along the guidewires to define and remove the anterior resection wedge. Once the wedge was removed, the osteotomy gap was manually closed to verify that the anterior cortex had fully compressed. A 4 mm drill bit was then placed in the most proximal and medial aspect of the tibia. This served as a joystick to control the rotation of the proximal segment, allowing for coronal‐plane manipulation. Using this tool, the proximal tibia was rotated such that the medial tibial plateau was lowered relative to the lateral side, thereby correcting the valgus deformity.

An alignment rod was used intraoperatively to assess the correction achieved. If the mechanical axis remained insufficiently corrected, an additional resection would be performed along the medial aspect of the distal osteotomy border. This would facilitate further coronal‐plane rotation and improved alignment. Final fixation was achieved using a compressive screw inserted from the distal and lateral tibial cortex towards the proximal and medial cortex, effectively locking the osteotomy in its corrected position. A medial locking plate was then applied to secure the construct and ensure stability during healing.

### Post‐operative course

**Figure 7:**
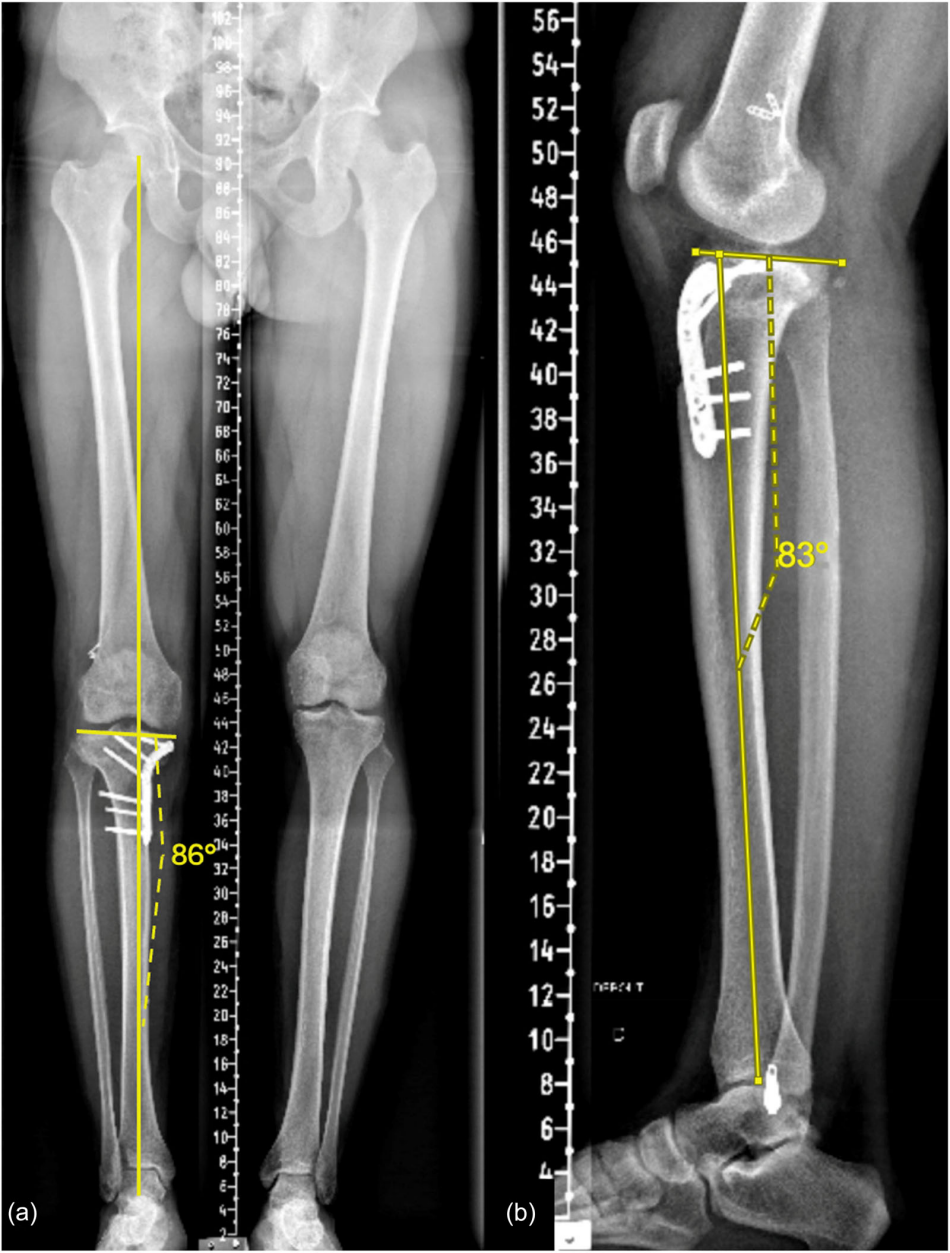
Post‐operative radiographs 12 months after biplanar medial closing wedge high tibial osteotomy. The medial proximal tibial angle was corrected to 86°, and the posterior tibial slope was corrected to 7°.

Post‐operatively, the patient was placed in a PCL brace, which was maintained for 6 weeks to protect the osteotomy site and control knee motion. NWB was advised for the first 3 weeks, followed by an additional 3 weeks of PWB with gradual progression based on clinical and radiographic healing. Return to work was permitted at approximately 2 months post‐operatively. A gradual return to sport‐specific activities, including swimming, running and cycling, was initiated at 3 months, with continued focus on range of motion, strength and neuromuscular control (Figure 7).

## CASE 3: BIPLANAR CORRECTION—MOW‐HTO WITH DIFFERENTIAL OPENING TO CORRECT VARUS AND PTS

### Clinical history

A 34‐year‐old male presented with medial right knee pain with running, as well as instability with cutting/pivoting sports. Past surgical history included two previous ACL reconstructions on the same knee, ACL reconstruction with hamstring autograft 12 years prior, and revision ACL reconstruction with patellar tendon autograft and a partial medial meniscectomy 7 years prior. On examination, there was medial joint line tenderness, a positive Lachman and a positive pivot shift testing.

### Radiographs and planning of correction

**Figure 8:**
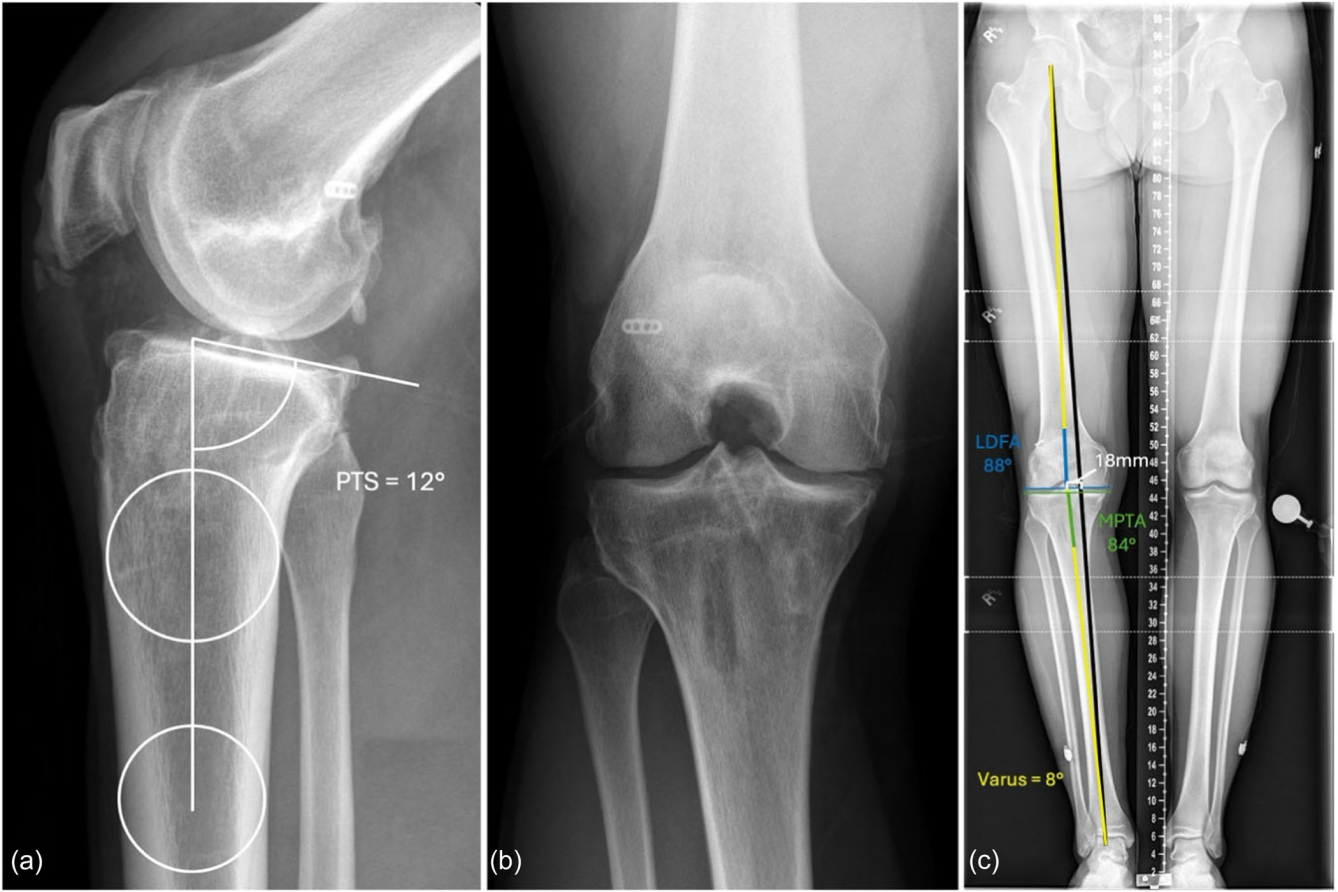
Weight‐bearing lateral radiograph of the right knee (a), 45° flexion posteroanterior (PA) radiograph (b) and long cassette radiograph (c). The long cassette radiograph demonstrates an MPTA of 84°, LDFA of 88°, mechanical axis deviation of 18 mm and 8° overall varus (c). The lateral radiograph (a) shows a posterior tibial slope (PTS) of 12° and evidence of patella baja. The weight‐bearing 45° PA flexion radiograph demonstrates Kellgren–Lawrence Grade II osteoarthritis of the medial compartment (b). LDFA, lateral distal femoral angle; MPTA, medial proximal tibial angle.

Standard weight‐bearing radiographs demonstrated Kellgren–Lawrence Grade II osteoarthritis (OA) of the medial compartment, patellofemoral joint degeneration with joint space narrowing, and osteophytes at the superior pole of the patella. The PTS measured 12° on the lateral view. Long cassette weight‐bearing anteroposterior (AP) radiographs revealed an 8° mechanical varus with 18 mm of mechanical axis deviation (MAD), an MPTA of 84°, JLCA of 2° and an LDFA of 88° (Figure 8).

### Assessment and plan

The clinical picture reflects post‐meniscectomy and post‐ACL reconstruction OA, with symptoms consistent with medial compartment overload, ACL insufficiency, and a primarily tibial‐based varus bony malalignment. A MOW‐HTO was proposed to offload the symptomatic degenerative medial compartment. With a modest PTS of 12° in the setting of two previous failed ACL reconstructions, it was felt that the PTS could be corrected with differential AP opening of the MOW. To address the symptomatic instability, a revision ACL reconstruction would be performed in a second stage. An important consideration in this case is that the patient has borderline patella baja as seen on the lateral knee radiographs (Figure 8a). In planning the osteotomy, the surgeon should avoid creating further patella baja.

### Surgical planning

**Figure 9:**
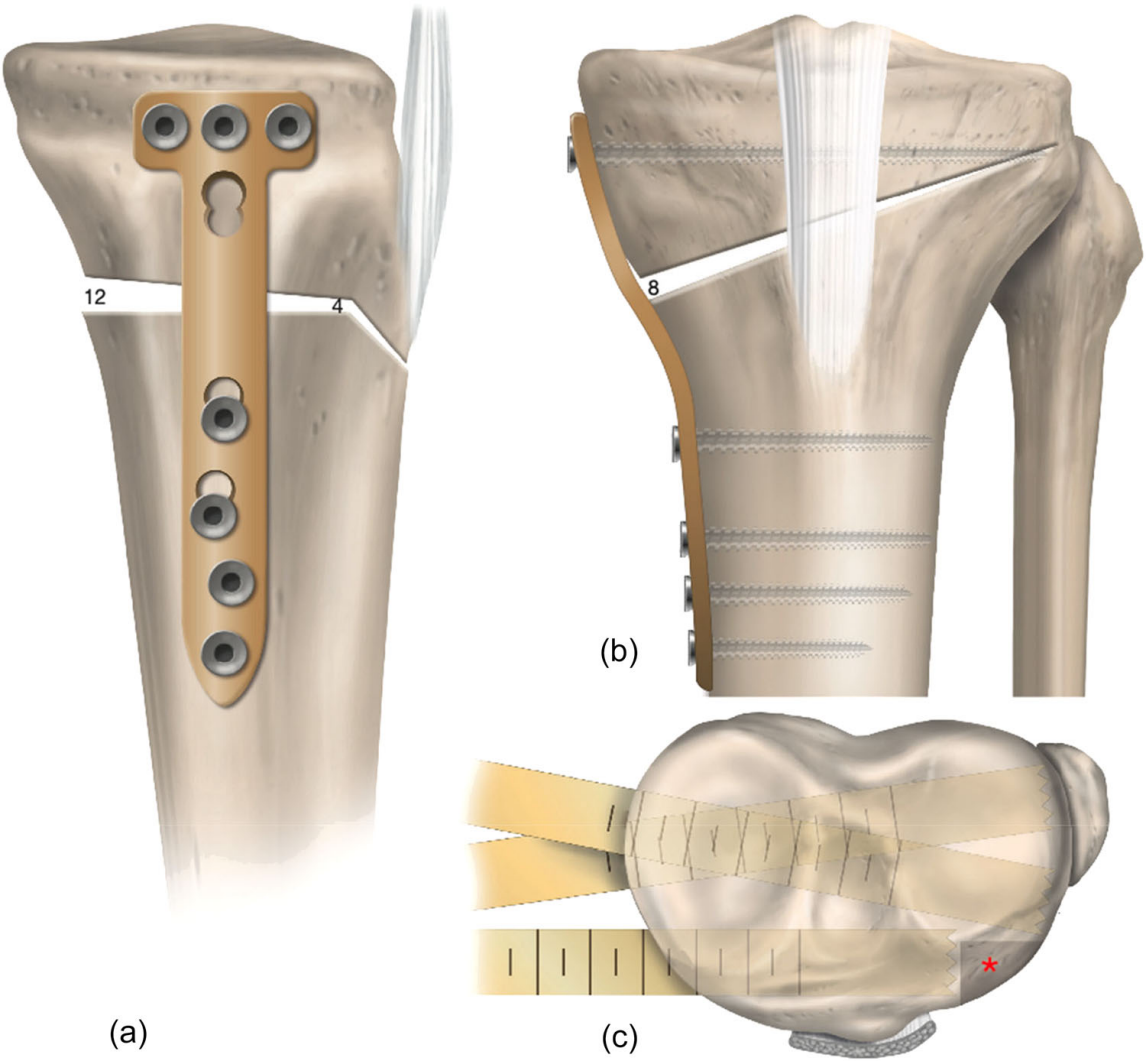
Schematic drawing of a medial opening wedge high tibial osteotomy with differential opening for concurrent correction of varus and posterior tibial slope. (a) Lateral view: the osteotomy gap is opened in a differential manner from posterior (12 mm) to anterior (4 mm) with an 8 mm differential. An infratubercle step cut is used to allow greater posterior opening while minimizing the risk of patella baja. (b) Anteroposterior view: the central gap (8 mm) is visible, and the osteotomy cut is directed towards the tip of the fibular head while leaving an adequate lateral hinge. (c) Maintaining the anterolateral hinge intact is crucial to enable differential opening (red asterisk).

In this case, an MOW‐HTO to correct both varus and PTS was planned using differential opening of the MOW. To correct the varus to 50% of the tibial plateau width and a planned post‐operative MPTA of 90°, an 8 mm opening wedge was planned centrally in the osteotomy (Figure 9). To correct PTS, a 4 mm opening was planned anteriorly and 12 mm opening was planned posteriorly within the opening wedge. The authors aimed for an 8 mm differential opening from anterior to posterior to attempt concurrent PTS correction. To allow for greater differential opening and reduce the risk of worsening patella baja, an infratubercle step cut was planned.

### Surgical steps and pearls

**Figure 10:**
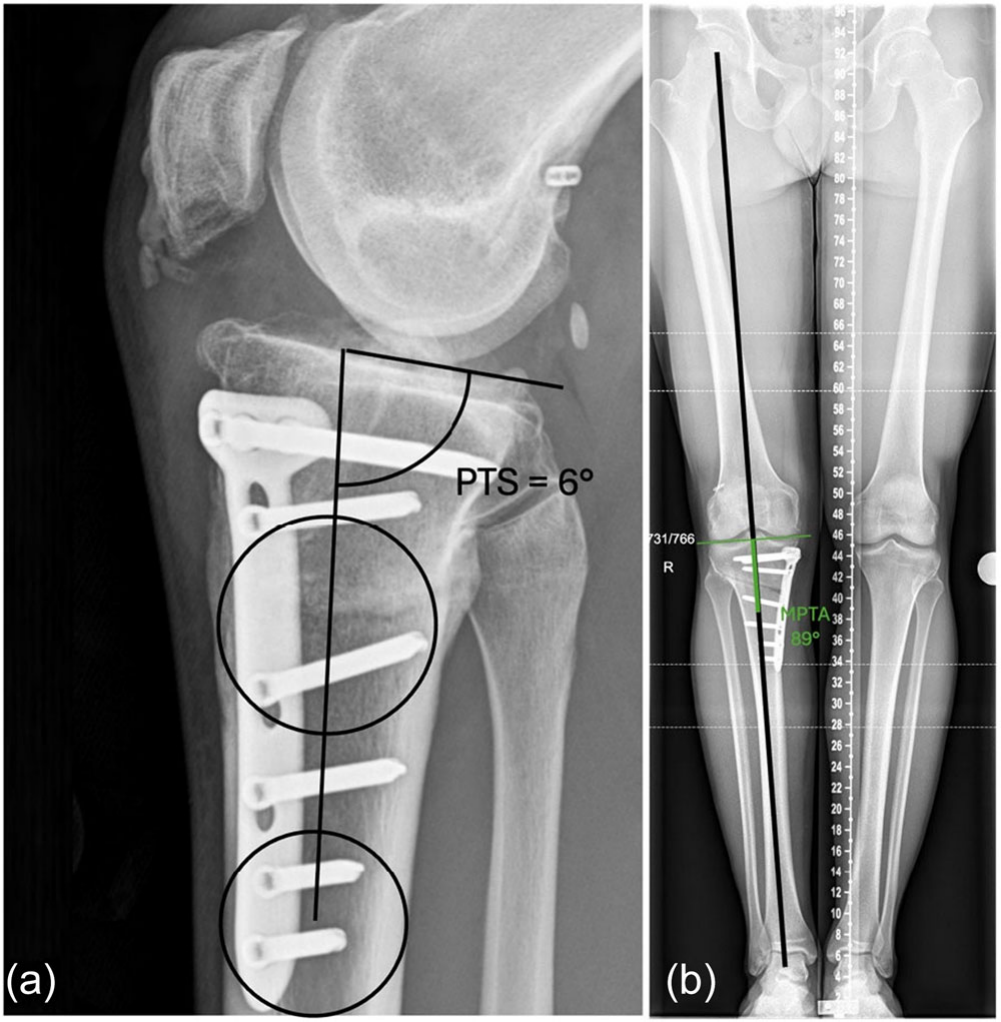
Post‐operative weight‐bearing lateral radiograph of the right knee (a) and long cassette radiograph (b) following medial opening wedge high tibial osteotomy with differential opening to correct both varus and posterior tibial slope (PTS). Long cassette radiograph shows correction of the medial proximal tibial angle (MPTA) to 89°, and the overall weight‐bearing axis to 50% of the tibial plateau width (b). Lateral radiograph shows a correction of the PTS to 6° (a).

An 8 cm anteromedial skin incision was used, extending from the joint line past the tibial tubercle. The medial collateral ligament was elevated, ensuring complete elevation posteriorly at the level of the planned osteotomy. Two K‐wires were placed beginning just above the level of the pes anserine tendons and aimed towards the tip of the proximal fibula. We ensured to leave an adequate anterolateral hinge during placement and confirmed the K‐wires were parallel to the joint line in the sagittal plane. The osteotomy was carried out using a reciprocating saw—parallel to the K‐wires posteriorly with an infratubercle step cut anteriorly (Figure 9) to prevent patella baja. Stacked osteotomes and a laminar spreader were used to obtain the desired correction—4 mm anterior, 8 mm central and 12 mm posterior in the MOW. The osteotomy was fixed with a medial locking plate. Post‐operative long cassette radiographs and true lateral radiographs of the right knee are displayed in Figure 10.

In our experience, this level of differential opening—where the anterior gap is 1/3 the posterior gap—is the maximum that can be achieved without risking hinge fracture. A laminar spreader is used posterior to the plate to maintain the wider opening, along with a combination of patient positioning (forced hyperextension) and/or temporary fixation methods (large fragment AO reduction forceps, K‐wires) during fixation. The literature suggests that gap differentials within 2 mm or where the anterior gap is 1/2 to 2/3 of the posterior gap are not likely to result in material PTS alteration during MOW‐HTO [24]. Furthermore, using the 1/3 rule in our experience does not lead to reliable slope reduction—we have observed reductions in degree of slope correction up to approximately 1/2 the differential. It is therefore a technique that, with current knowledge, should be considered only in cases of borderline high PTS such as this patient.

## CASE 4: BIPLANAR CORRECTION—SINGLE‐STAGE INFRATUBEROSITY ACW‐HTO AND MOW‐HTO TO CORRECT VARUS AND PTS

### Clinical history

A 32‐year‐old male presented with medial left knee pain and recurrent instability, unable to participate in sports. Past surgical history included one previous ACL reconstruction 11 years prior with hamstring autograft. Three years prior to presentation, he sustained failure of his ACL graft while playing soccer, but decided against revision ACL surgery at that time due to employment and family commitments. Clinical examination demonstrated medial joint line tenderness, a positive Lachman, and a positive pivot shift test.

### Radiographs and planning of correction

**Figure 11:**
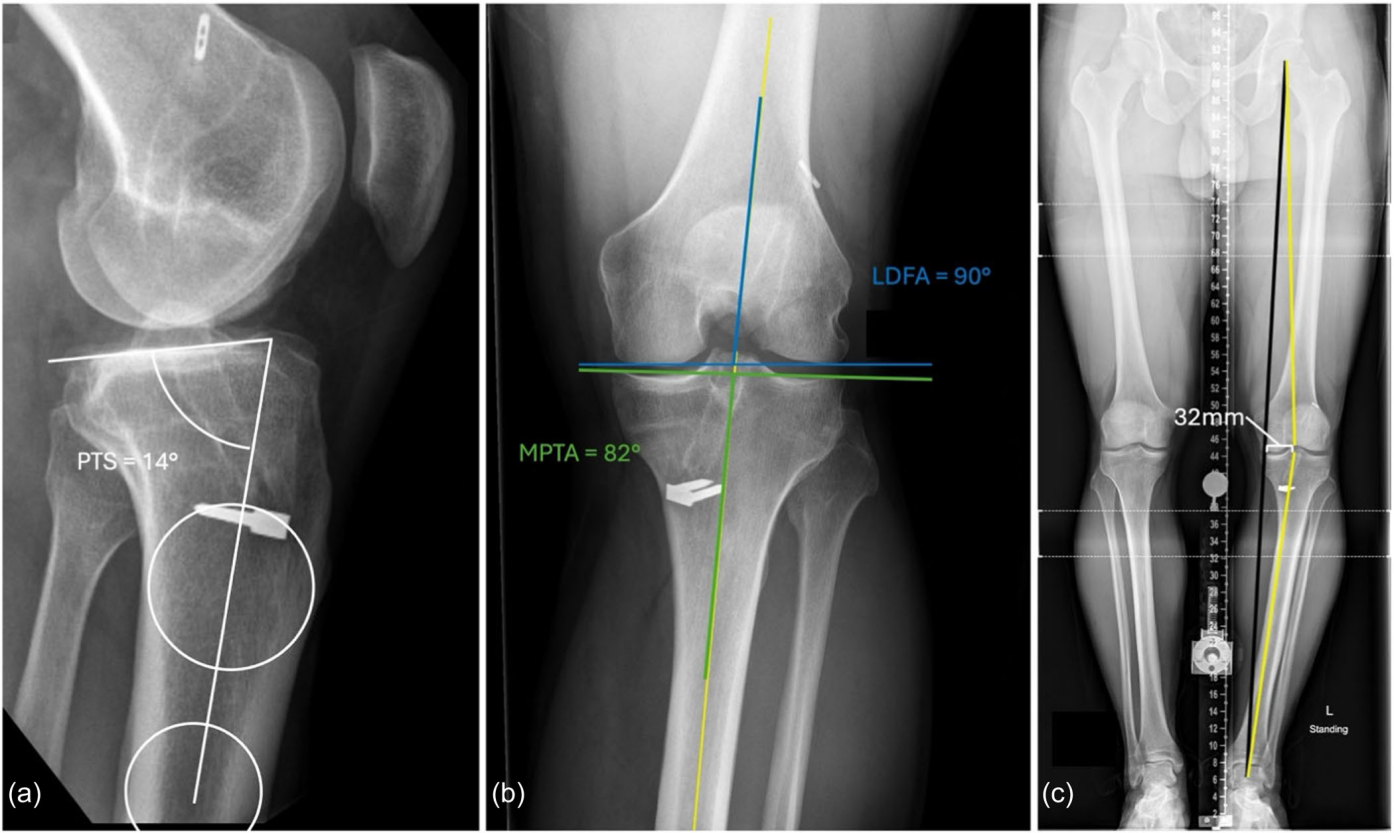
Weight‐bearing lateral radiograph (a), anteroposterior (AP) radiograph (b) and long cassette radiograph (c) focusing on the left knee. The AP radiograph and long cassette radiograph (b, c) show varus alignment with a medial proximal tibial angle (MPTA) of 82°, a lateral distal femoral angle (LDFA) of 90° and a mechanical axis deviation of 32 mm. The true lateral radiograph (a) of the left knee demonstrates a posterior tibial slope (PTS) of 14°.

Standard weight‐bearing radiographs of the left knee demonstrated an overall varus alignment and mild medial joint space narrowing (Figure 11). Long cassette weight‐bearing AP radiographs revealed a mechanical varus with an MPTA of 82°, an LDFA of 90° and a MAD of 32 mm. The PTS measured 14° on the lateral view, indicating elevated PTS (Figure 11). An MRI was completed of the left knee, which demonstrated a 10 × 8 mm full‐thickness chondral defect in the central weight‐bearing zone of the medial femoral condyle, a torn ACL graft, a vertical and anterior femoral tunnel out of the way of future ACL reconstruction, and a posterior non‐anatomic tibial tunnel of 10 mm diameter.

### Assessment and plan

The clinical picture reflects medial compartment overload with an overall varus alignment, a chondral defect in the weight‐bearing zone of the medial femoral condyle, and chronic ACL insufficiency following previous ACL reconstruction with a high PTS. The varus deformity is primarily tibial‐based, warranting consideration of a realignment HTO to offload the symptomatic medial compartment in the setting of medial compartment overload and a full‐thickness chondral defect of the medial femoral condyle. A PTS of 14° in the setting of chronic ACL insufficiency is too elevated to consider differential opening as in the previous case, with similar confounding of varus driven by the tibial deformity. To address this problem, the authors planned for both an MOW‐HTO to correct varus and an infratuberosity ACW‐HTO to correct elevated PTS in a single stage. Bone grafting of the tibial tunnel (not wide by definition, but too posterior for future anatomic ACL reconstruction) would also be performed in this stage, as well as a microfracture of the chondral defect in the medial femoral condyle (with a more advanced cartilage procedure during future ACL reconstruction if unsuccessful).

### Surgical planning

**Figure 12:**
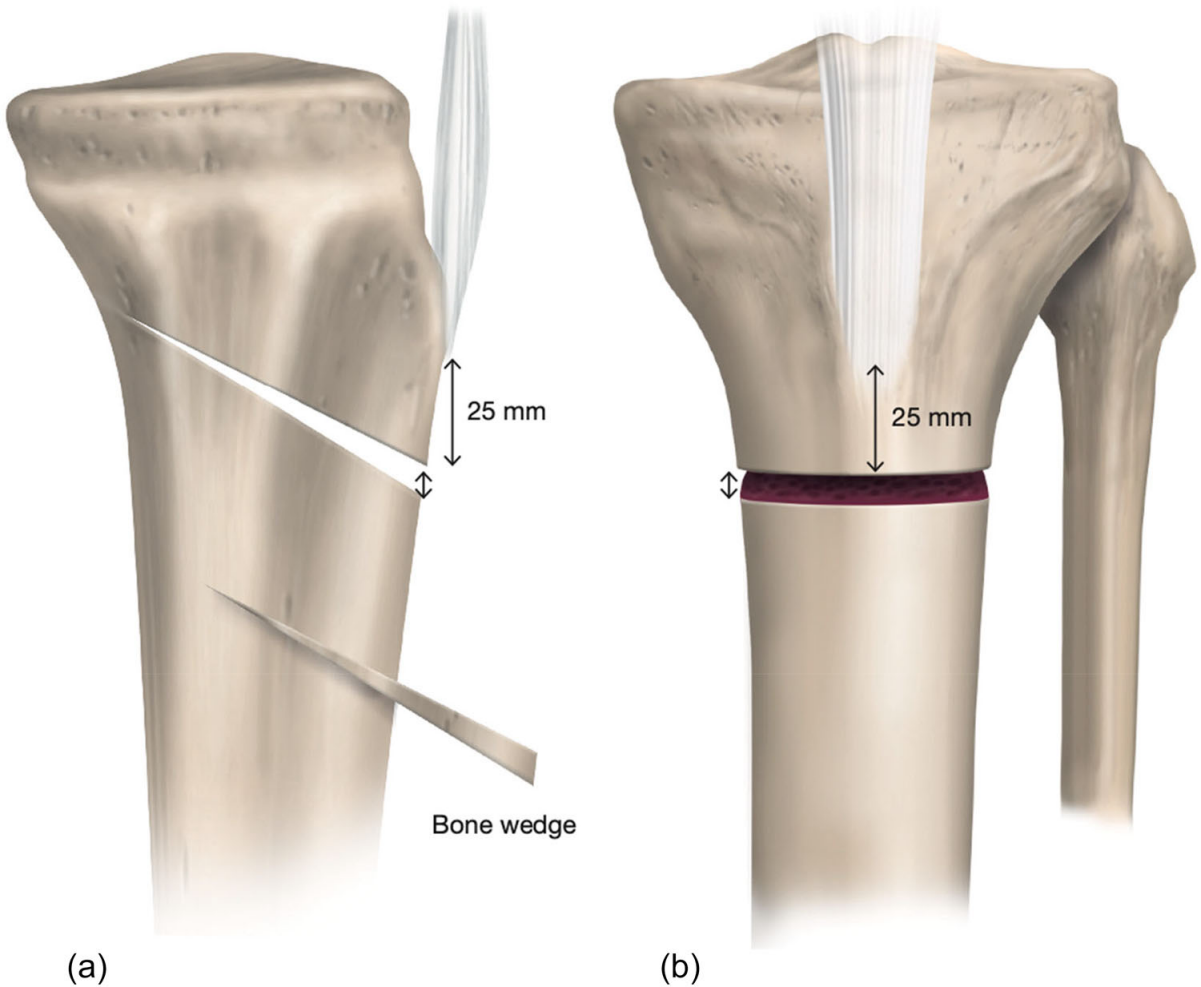
Lateral (a) and anteroposterior (b) views illustrate an infratuberosity anterior closing wedge high tibial osteotomy. The starting point for the osteotomy is 25 mm distal to the tibial tubercle.

**Figure 13:**
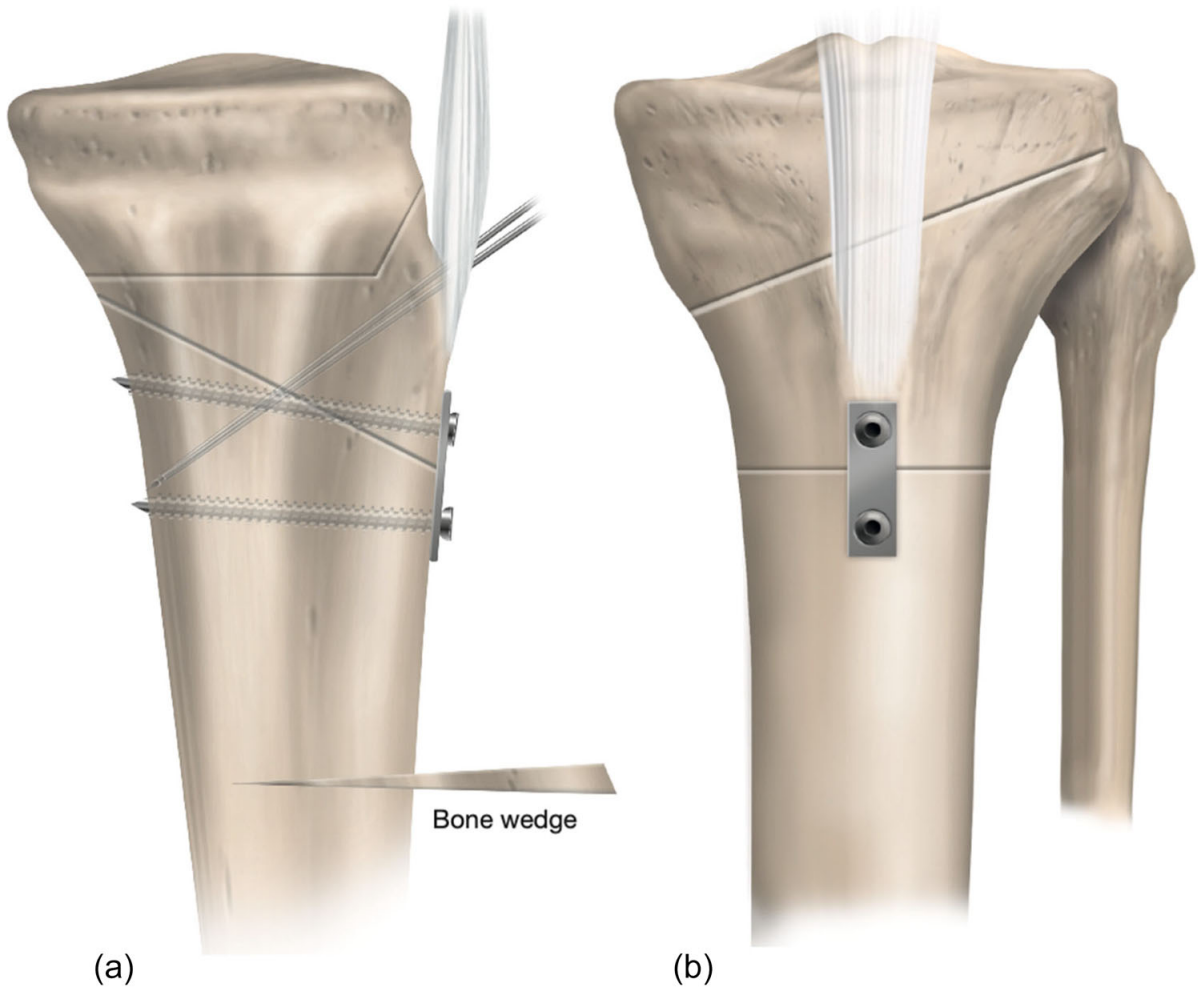
Infratuberosity anterior closed‐wedge high tibial osteotomy (ACW‐HTO), fixed using a one‐third tubular plate and two small‐fragment bicortical screws placed distal to the tibial tubercle (a). Prior to opening the medial opening wedge HTO, two 2‐mm K‐wires are inserted from the tibial tubercle, crossing perpendicular to the ACW osteotomy, to provide additional stability and prevent displacement (b). On the anteroposterior view, the osteotomy cut is aimed towards the tip of the fibular head leaving an intact lateral hinge.

**Figure 14:**
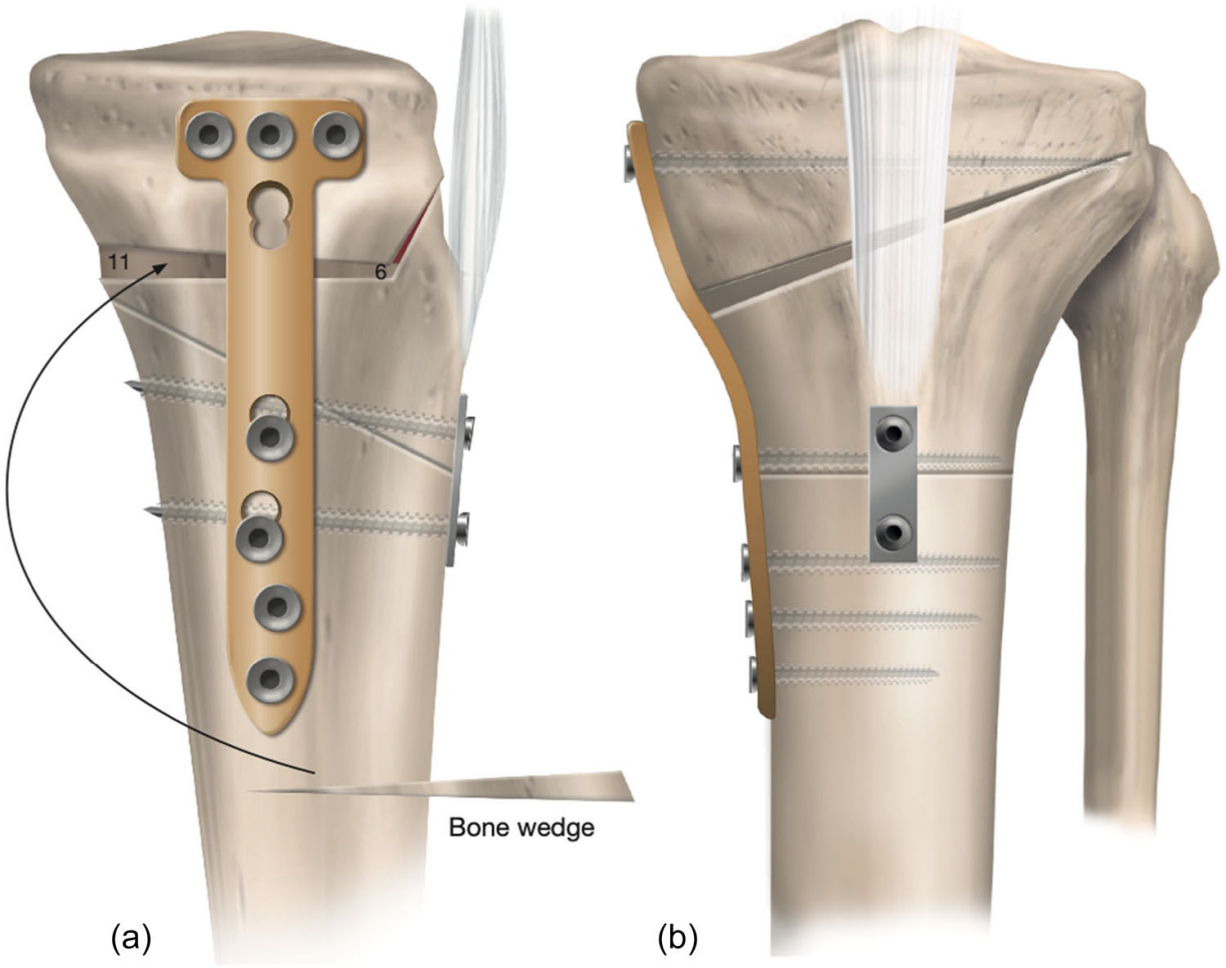
(a) Medial opening wedge high tibial osteotomy (MOW‐HTO) with differential opening: 6 mm anteriorly, 9 mm centrally and 11 mm posteriorly. The harvested autologous proximal tibial bone wedge from the anterior closing wedge HTO is transferred to the MOW‐HTO gap. (b) Both osteotomy sites are stabilized with a medial locking plate, and the two K‐wires are removed.

In this case, both an infratuberosity ACW‐HTO (Figure 12) and a separate higher MOW‐HTO (Figure 13) were used to correct both varus and PTS. To correct the varus to 50% tibial plateau width and a MPTA of 90°, a 9 mm opening wedge was planned centrally in the osteotomy. The autologous wedge harvested from the ACW‐HTO site was used to augment the MOW‐HTO gap (Figure 14). To avoid risking increasing the PTS from the MOW‐HTO, a 6 mm opening was planned anteriorly and an 11 mm opening was planned posteriorly in the MOW. For correction of the PTS, a separate infratuberosity ACW‐HTO was planned with a closing wedge (8° correction), aiming for a final PTS of ~6°.

### Surgical steps and pearls

The procedure began with a left knee arthroscopy and microfracture of the <1 cm^2^ Grade IV chondral lesion in the weight‐bearing zone of the medial femoral condyle. Following this, the open procedure was carried out through a 10‐cm anteromedial skin incision from the joint line distal to the tibial tubercle. Tunnel grafting of the ACL tibial tunnel with a 10‐mm pre‐fashioned allograft bone dowel was then performed after removing the previous staple, graft material, and suture from the tibial tunnel. Next, an infratuberosity ACW‐HTO planning for an 8° correction was performed over four K‐wires (two proximal and two distal), starting 25 mm below the tibial tubercle, and converging at the planned posterior hinge point in the sagittal plane (Figure 12). This osteotomy was closed through forced hyperextension and provisionally fixed with a one‐third tubular plate and two small fragment bicortical screws (Figure 13). A MOW‐HTO with differential opening was then performed above the ACW‐HTO—6 mm anteriorly, 9 mm centrally and 11 mm posteriorly within the opening wedge (Figure 14). This differential opening served to avoid an increase in the PTS during MOW correction. It is important to note that prior to opening the MOW‐HTO, two 2‐mm K‐wires were placed perpendicular to the ACW osteotomy site for further security during opening (Figure 13). After distracting the MOW, the autograft bone wedge removed during the ACW‐HTO was transferred to the MOW‐HTO. Both osteotomy sites were then spanned with a medial locking plate (Figure 14).

**Figure 15:**
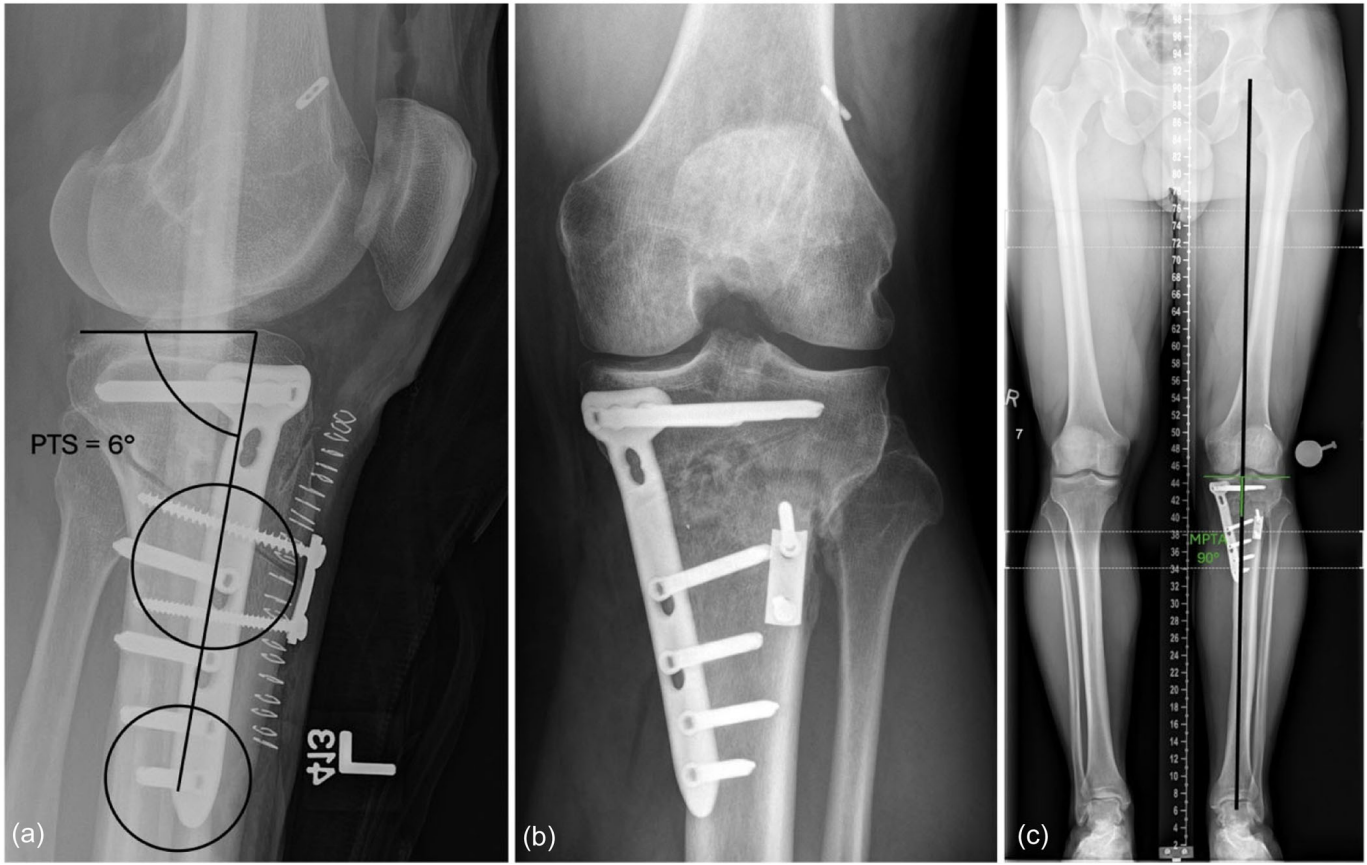
Immediate post‐operative lateral radiograph (a) and 3‐month post‐operative anteroposterior (AP) radiograph (b) of the left knee following combined medial opening wedge high tibial osteotomy (MOW‐HTO) and infratuberosity anterior closing wedge HTO to correct both varus and posterior tibial slope (PTS). Post‐operative lateral radiograph of the left knee shows a correction of the PTS to 6° (a). Three‐month post‐operative long cassette radiograph shows correction of the medial proximal tibial angle to 90°, and the overall weight‐bearing axis to 50% of the tibial plateau width (c).

Immediate post‐operative radiographs and 3‐month post‐operative long cassette standing AP radiographs of the left knee are displayed in Figure 15.

## OUTCOMES

### Frontal plane correction

Outcomes following HTO have been consistently favourable when carefully considering patient selection and surgical technique. In the setting of isolated varus deformity, MOW‐HTO has demonstrated high rates of functional improvement and pain relief, with approximately 76%–87% of patients returning to sport or work post‐operatively [10, 51]. Survival rates of the native knee at 10 years following MOW‐HTO are reported between 70% and 90.4%, particularly among younger, active individuals [1, 16]. Peak clinical benefits are typically achieved within 12 months and can be maintained long‐term if proper mechanical alignment is restored [1, 44]. Similarly, LCW‐HTO has demonstrated good to excellent results, with a mean survivorship of 12.6 years and a conversion rate to TKA of nearly 15% [14, 39]. Historically, the direct bone‐to‐bone healing was popularized in 1965, and LCW‐HTO offers the advantage of direct bone‐to‐bone healing, promoting structural stability [7]. However, it carries a slightly higher risk of peroneal nerve palsy and a higher revision rate to TKA compared to MOW‐HTO [15, 39]. Nonetheless, contemporary arthroplasty outcomes following prior HTO have significantly improved, mitigating concerns over subsequent TKA following HTO [60].

In valgus knee deformities, MCW HTO has demonstrated promising results, yet it is performed less frequently than MOW‐HTO [53]. Over 70% of patients achieved good or very good clinical outcomes at the 7‐year follow‐up, with more than 90% experiencing significant pain reduction [52]. At nearly 10 years post‐operatively, approximately 19% required conversion to TKA [39, 56]. While less commonly performed, lateral opening wedge (LOW) HTO for valgus correction has also demonstrated favourable outcomes. One study reported significant improvements in clinical scores, radiographic parameters, and gait analysis at an average follow‐up of 52 months, with only 10% of patients undergoing conversion to TKA [5].

### Sagittal plane correction

Sagittal plane correction has emerged as an essential adjunct in the setting of ligamentous knee instability. ACW‐HTO aimed at reducing PTS is mainly used in revision ACL reconstruction for patients with an elevated PTS (>12°) and persistent instability [23, 33, 42]. PTS reduction significantly reduces ACL‐graft forces and improves anterior knee stability [4, 21, 31]. Patients undergoing combined revision ACL reconstruction and ACW‐HTO have demonstrated restoration of knee stability and return to preoperative activities [11, 59, 63]. Conversely, AOW‐HTO has been proposed to manage chronic PCL deficiency in the setting of a decreased PTS (<4°), improving posterior tibial translation and graft protection [23]. Although long‐term clinical outcome data for AOW‐HTO remain limited, initial reports suggest restoration of biomechanics and knee stability [23, 67].

### Biplanar correction

Biplanar correction illustrates a valuable surgical treatment option for successful realignment in both the coronal and sagittal planes, but literature on outcomes is scarce. Technical notes and case reports have been published with good results but with small sample sizes [6, 13, 20, 30, 34, 43, 72]. One study evaluated the effect of combined MOW‐HTO with PTS modification in arthritic knees with extension deficit. In total, 18 patients were followed for a mean of 1.5 years and showed favourable clinical outcomes [72]. Similar results were shown in a cohort of six patients who underwent modified MOW‐HTO to simultaneously correct varus and PTS deformities in ACL‐deficient knees. At a mean follow‐up of 21 months, all six patients returned to preoperative activity levels [13]. Another study investigated the precision of a patient‐specific cutting guide and plate fixation system in varus malalignment [71]. The authors demonstrated that precise correction of the varus deformity could be achieved while maintaining the PTS. Therefore, extrapolating on their findings, PSI in the setting of biplanar correction may be a viable option, allowing for a deeper understanding of the 3D position of the planned hinge point [71]. In summary, there is a need for future outcome studies with larger cohorts and standardized surgical techniques before definite conclusions can be made regarding biplanar corrections.

## CONCLUSION

Biplanar HTO is a knee‐preserving surgical option for a small cohort of patients with complex knee deformities involving both the coronal and sagittal planes. Precise preoperative planning and meticulous surgical execution are essential to address these biplanar malalignments effectively. This review serves as a guide for orthopaedic surgeons, highlighting key considerations if biplanar HTO is considered and serves as a practical guide in complex cases.

## AUTHOR CONTRIBUTIONS

**Matthieu Ollivier**: Conceptualization; writing—review and editing. **Volker Musahl**: Conceptualization; writing—review and editing. **Tyler M. Hauer**: Conceptualization; methodology; writing—original draft preparation; writing—review and editing. **Romed P. Vieider**: Methodology; writing—original draft preparation; writing—review and editing. **David Wasserstein**: Methodology; writing—review and editing.

## CONFLICTS OF INTEREST STATEMENT

Matthieu Ollivier is consultant and receive royalties from Stryker, Newclip Technics. Volker Musahl declares educational grants, consulting fees and speaking fees from Smith & Nephew plc, educational grants from Arthrex and DePuy/Synthes, is a board member of the *International Society of Arthroscopy, Knee Surgery and Orthopaedic Sports Medicine* (ISAKOS), and deputy editor‐in‐chief of *Knee Surgery, Sports Traumatology, Arthroscopy* (KSSTA). David Wasserstein declares that he is a consultant for ConMed and that a portion of the training and research grant was paid to his institution. The remaining authors declare no conflicts of interest.

## ETHICS STATEMENT

The ethics statement is not available.

## Data Availability

The data that support the findings of this study are available on request from the corresponding author. The data are not publicly available due to privacy or ethical restrictions.
